# Álvaro Moncayo Medina 1941-2019

**Published:** 2020-03-30

**Authors:** Mario Ivan Ortiz, Ximena Moncayo, Jorge Alberto Molina

**Affiliations:** 1 Centro de Investigaciones en Microbiología y Parasitología Tropical, Universidad de los Andes, Bogotá D.C., Colombia Universidad de los Andes Centro de Investigaciones en Microbiología y Parasitología Tropical Universidad de los Andes Bogotá D.C Colombia

El pasado 23 de diciembre de 2019 falleció en Girardot, Cundinamarca, el médico epidemiólogo Álvaro Moncayo Medina. Su amplia y destacada trayectoria en el campo de la Epidemiología y la Salud Pública, así como su importante influencia en las nuevas generaciones de investigadores, ameritan estas palabras de homenaje al maestro y amigo.

El doctor Moncayo nació en San Juan de Pasto, Nariño, el 14 de mayo de 1941. Se graduó como bachiller del Colegio San Francisco Javier de Pasto en 1959 y completó sus estudios universitarios en la Facultad de Medicina de la Pontificia Universidad Javeriana de Bogotá en 1969. Posteriormente, en 1970, hizo su maestría en Salud Pública y una especialización en Epidemiología en la Universidad de Antioquia. En 1972 adelantó estudios de especialización en Epidemiología de Enfermedades Transmisibles en los *Centers for Disease Control and Prevention* (CDC) de Atlanta, Estados Unidos.

A partir de 1976, la carrera del doctor Moncayo se desarrolló en la Organización Mundial de la Salud (OMS), donde ocupó diversos cargos como consultor en Epidemiología en Argentina, para luego hacer parte del *Special Programme for Research and Training in Tropical Diseases* (TDR) de esta entidad en Ginebra. En la OMS participó activamente en diferentes actividades, pero sin perder nunca de vista la importancia de las enfermedades tropicales para la salud pública en el trópico. Prueba de ello es que desde allí apoyó y colaboró en el desarrollo de programas de control, prevención y manejo de enfermedades tropicales, en especial, programas enfocados en la enfermedad de Chagas, la leishmaniasis y la enfermedad del sueño. Gracias a estos programas se impulsaron proyectos que buscaban mejorar el diagnóstico y el control vectorial de los transmisores de estas enfermedades en diferentes países de Latinoamérica y África. Uno de sus grandes logros fue el establecimiento de los programas de control vectorial de la enfermedad de Chagas en diferentes países de Suramérica.

En el 2001, después de obtener su pensión de la OMS, decidió regresar a su amada Colombia y, por su interés constante de transmitir las experiencias adquiridas, se vinculó como investigador *ad honorem* al Centro de Investigaciones en Microbiología y Parasitología Tropical (CIMPAT) de la Universidad de los Andes. Aquí logró transmitir su pasión por la Epidemiología y la Salud Pública a alumnos de pregrado y posgrado de los programas de Biología, Microbiología y Medicina, entre otras carreras. Durante su estadía en el CIMPAT merece mención especial su aporte a la organización y la dirección de los seminarios semanales del laboratorio.

A lo largo de su carrera, el doctor Moncayo publicó sus estudios en diversas revistas científicas como *The Lancet,* la *International Journal of Epidemiology,* los *Annals of Tropical Medicine and Parasitology,* el *CDC Morbidity and Mortality Weekly Report,* la *Infection Genetics and Evolution* y *Biomédica,* entre otras. A ello se suman los libros y más de once capítulos de libros, editados en Colombia y en el exterior. Además, es muy importante resaltar que, en su interés por divulgar sus ideas y opiniones más allá de los ambientes académicos, publicó en diversas ocasiones en medios internacionales tan importantes como *Le Monde Diplomatique.*

Como es natural, una carrera académica e investigativa tan comprometida obtuvo el reconocimiento con diversos premios, distinciones y cargos, entre ellos, el Premio Carlos Chagas otorgado por el Instituto Oswaldo Cruz de Río de Janeiro (2000); su nombramiento como Académico Correspondiente Extranjero de la Academia Nacional de Medicina de Buenos Aires (1997), como Académico Honorario Extranjero de la Academia Nacional de Medicina del Brasil (2005), y como Secretario General y Vicepresidente de la Academia Nacional de Medicina de Colombia (2006 y 2014); la Condecoración Samper-Martínez otorgada por el Instituto Nacional de Salud de Colombia (2007) y su designación como miembro del comité asesor de *Disease Control Priorities 3* de la Fundación Gates (2012), entre otros. Asimismo, fue miembro de diferentes sociedades científicas internacionales y nacionales como la *Epidemiological Association,* la *Royal Society of Tropical Medicine and Hygiene,* la *Societé Suisse de Medicine Tropicale* y la Sociedad Colombiana de Parasitología y Medicina Tropical.

En su vida personal, el doctor Moncayo fue un escéptico por naturaleza, un apasionado humanista, voraz lector, amante de la mitología griega, el cine y la buena cocina y dueño de una cultura que abarcaba áreas como el arte, la historia, la música, la ciencia y la literatura. Esta pasión por la cultura y el conocimiento sin fronteras la trasmitió de manera generosa a sus alumnos y amigos, siempre dispuesto a prestar los libros de su biblioteca personal para motivar la lectura y a participar en el intercambio de ideas a través de discusiones y conversaciones acompañadas de un buen café.

Sin duda alguna, sus alumnos y amigos vamos a extrañar a este ciudadano del mundo que, con su visión global y culta de la realidad, nos permitió no solo expandir nuestros conocimientos, sino también, matricularnos de manera más activa en la lucha por un mundo más justo e igualitario.

Compartimos con sus hermanas, sus sobrinos y sus cuñados, el sentimiento de pérdida de un ser humano excepcional que contribuyó con su trabajo al mejoramiento de la calidad de vida de miles de personas y que dejó en alto el nombre de nuestro país.

Quienes conocimos al doctor Moncayo queremos despedirlo citando uno de sus versos preferidos de los sonetos del poeta Eduardo Carranza, que recitaba constantemente: "Salvo mi corazón, todo está bien".

